# The Circadian Clock Regulates Auxin Signaling and Responses in *Arabidopsis*

**DOI:** 10.1371/journal.pbio.0050222

**Published:** 2007-08-07

**Authors:** Michael F Covington, Stacey L Harmer

**Affiliations:** Section of Plant Biology, College of Biological Sciences, University of California Davis, Davis, California, United States of America; Max Planck Institute for Developmental Biology, Germany

## Abstract

The circadian clock plays a pervasive role in the temporal regulation of plant physiology, environmental responsiveness, and development. In contrast, the phytohormone auxin plays a similarly far-reaching role in the spatial regulation of plant growth and development. Went and Thimann noted 70 years ago that plant sensitivity to auxin varied according to the time of day, an observation that they could not explain. Here we present work that explains this puzzle, demonstrating that the circadian clock regulates auxin signal transduction. Using genome-wide transcriptional profiling, we found many auxin-induced genes are under clock regulation. We verified that endogenous auxin signaling is clock regulated with a luciferase-based assay. Exogenous auxin has only modest effects on the plant clock, but the clock controls plant sensitivity to applied auxin. Notably, we found both transcriptional and growth responses to exogenous auxin are gated by the clock. Thus the circadian clock regulates some, and perhaps all, auxin responses. Consequently, many aspects of plant physiology not previously thought to be under circadian control may show time-of-day–specific sensitivity, with likely important consequences for plant growth and environmental responses.

## Author Summary

Most higher organisms, including plants and animals, have developed a time-keeping mechanism that allows them to anticipate daily fluctuations of environmental parameters such as light and temperature. This circadian clock efficiently coordinates plant growth and metabolism with respect to time of day by producing self-sustained rhythms of gene expression with an approximately 24-h period. One of the major contributors in specifying spatial patterns of plant growth and development is auxin, a hormone essential for nearly all stages of plant development. Auxin also helps the plant orient itself properly in response to environmental cues such as light, gravity, and water. We have now found circadian-regulated expression of components from nearly every step in the auxin-signaling pathway, from synthesis to response. We demonstrate the relevance of this observation by showing that plants have differential sensitivity to auxin at different times of day: the clock controls plant sensitivity to auxin at both the level of transcription and stem growth. Our work demonstrates an intimate connection between the clock- and auxin-signaling pathways, and suggests that other auxin-regulated processes may also be under circadian control.

## Abstract 3

The circadian regulation of auxin responses suggests that many aspects of plant physiology not previously thought to be under circadian control may show time-of-day-specific sensitivity.

## Introduction

Plants, which as sessile organisms are intimately tied to their environment, have evolved many ways to deal with changing local conditions. One coping mechanism is the circadian oscillator or clock, which produces self-sustained rhythms with an approximately 24-h period. It is often suggested that the clock provides an adaptive advantage by allowing organisms to anticipate regular changes in the environment and temporally separate incompatible metabolic events [1]. The importance of these rhythms has in fact been demonstrated in both phytoplankton and higher plants: organisms that have an internal clock period matched to the external environment possess a competitive advantage over those that do not [2,3].

At its simplest, a circadian system consists of input pathways that entrain the clock, the core oscillator or central clock itself, and clock output pathways. Many components of the plant circadian system have been identified in recent years, and the relationships between them are now being explored [4]. Similar clock genes are found in both monocots and dicots; however, the components of the plant central clock are not conserved with those of fungi and animals [5–7]. Although the components differ, the central oscillators of higher plants, animals, and fungi seem to be composed of similar interlocking transcription/translation feedback loops [8–10].

Another conserved characteristic of circadian clocks is their ability to modulate responses to stimuli depending upon the time of day, a phenomenon called “gating.” For example, a light pulse given during the subjective day induces expression of the photosynthesis gene *CHLOROPHYLL A/B BINDING PROTEIN 2 (CAB2),* a response that is absent following a light pulse during the subjective night [11]. Gating by the clock thus may restrict plant responsiveness to various stimuli to physiologically appropriate times of day. The growth advantage provided by the clock [3] might therefore be due to its ability to prevent plants from responding to untimely environmental inputs as well as to anticipate regular changes in the environment.

A substantial fraction of the *Arabidopsis* transcriptome is under circadian regulation [12–15], similar to what has been found in other model organisms [16–19]. The identification of clock-regulated genes may provide insight into the types of processes under circadian regulation. In addition, the promoters of clock-regulated genes may be used to control expression of firefly luciferase, providing a ready means to monitor the state of the circadian oscillator in living plants [20]. Other easily assayed circadian outputs in plants include rhythmic elongation of internodes [21] and embryonic stems (hypocotyls) [22]. Circadian rhythms modify many other aspects of plant physiology and development, ranging from cold adaptation to photosynthetic capacity to the transition between vegetative and reproductive growth [23].

A similarly pervasive role in physiology and development has been attributed to the plant hormone auxin [24], which was first discovered for its role in phototropism [25]. Subsequent work has revealed a central role for auxin in other directional growth responses such as gravitropism [26]. In addition to these roles in environmental responses, auxin is an important regulator of plant development. It plays an essential role during almost every stage of plant development, including embryogenesis, leaf and lateral root initiation, vascular patterning, and the establishment of apical dominance [27–30].

The most important auxin in higher plants is indole-3-acetic acid or IAA [24]. IAA and other auxins are synthesized primarily in shoot apical tissues and then actively transported towards the base of the plant [31]. Two very different types of proteins have been implicated in auxin perception. The first, auxin binding protein 1, has been suggested to act primarily in regulation of the cell cycle [32] and cell expansion [33]. The second type consists of a family of F-box proteins, transport inhibitor response 1 (TIR1) and three related proteins (auxin-signaling F-box protein 1–3 [AFB1–3]), which are important for transcriptional responses to auxin [34]. These F-box proteins are involved in the regulated degradation of a large family of transcriptional regulators, the Aux/IAA proteins. There are two additional closely related TIR1 homologues, AFB4 and AFB5, which are candidate auxin receptors [35].

Hundreds of genes are induced or repressed upon auxin treatment, and many of these contain an auxin-responsive element (AuxRE) in their promoter regions [36]. AuxREs are bound by a second large family of transcription factors called auxin response factors (ARFs), members of which can act either as transcriptional activators or repressors [37]. However, when auxin levels are low, ARF activity is inhibited by their heterodimerization with Aux/IAA proteins. As auxin levels increase, binding of auxin to the TIR1/AFB proteins promotes TIR1/AFB interactions with Aux/IAA proteins [34,35]. This leads to enhanced degradation of the Aux/IAAs, resulting in increased ARF activity. Thus the signal transduction pathway between auxin binding to TIR1 (and the related AFB proteins) and transcriptional responses is extraordinarily short.

Transcriptional profiling has previously been used both to identify genes that are under circadian regulation [12–14] and those that are repressed or induced in response to exogenous auxin [36,38]. Here we find that auxin-induced genes are more likely to be clock regulated than expected by chance, suggesting that the circadian clock might modulate auxin signaling. Indeed, we show that transcriptional responses to both endogenous and exogenous auxin are regulated by the circadian clock. In addition, we demonstrate that plant growth in response to exogenous auxin is gated by the clock. Went and Thimann noted decades ago that plant sensitivity to auxin varied with the time of day, with maximal sensitivity observed in the early morning hours [25]. Our findings explain their observation: the circadian clock modulates plant transcriptional and growth responses to auxin.

## Results

### Extensive Circadian Control of Auxin-Signaling Genes

**Figure 1:**
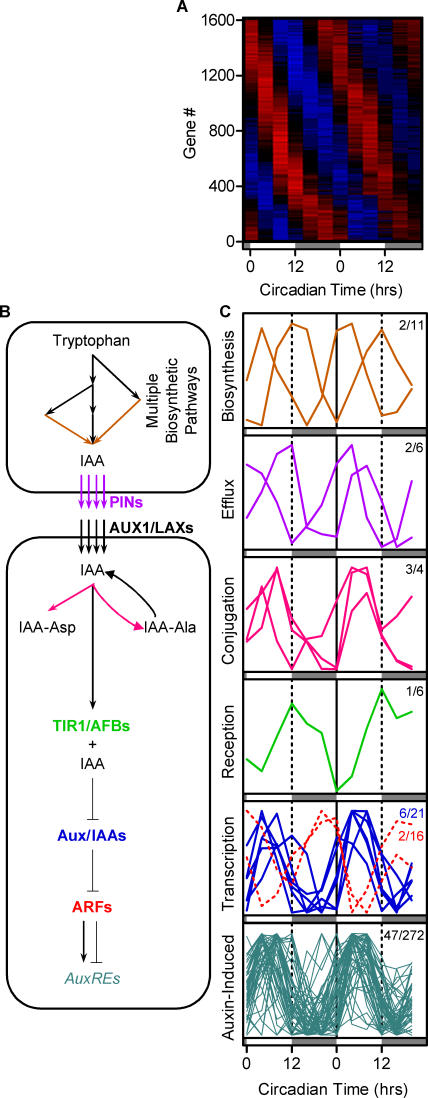
Many Genes in the Auxin-Signaling Pathway Are Circadian Regulated (A) Heatmap representation [74] of 1,610 circadian-regulated genes shows peak expression occurs at all phases. High expression is depicted in red and low expression in blue. (B) Schematic of the auxin-signaling pathway is presented (see Table S3 for the names of genes whose profiles are shown here) [31,34,37,39,42,43]. IAA is synthesized via tryptophan-independent (not shown) and multiple tryptophan-dependent pathways; two enzymes implicated in the final steps of the indole-3-acetaldoxime and indole-3-acetamide biosynthetic pathways are encoded by circadian-regulated genes. IAA, which undergoes polar transport throughout the plant, can be temporarily or permanently inactivated by conjugation to amino acids. Free IAA binds to the TIR1/AFB F-box proteins and facilitates their interaction with Aux/IAA proteins that are subsequently targeted for degradation. Therefore, in the presence of auxin, Aux/IAA proteins no longer prevent the ARF transcription factors from modulating expression of auxin-regulated genes. Steps with clock-regulated gene expression are color coded to match expression data in (C). (C) Normalized microarray expression data for circadian-regulated auxin-signaling [31,34,37,39,42,43] and auxin-induced [36] genes are shown. Genes have been grouped and color coded according to general function or class (see [B]). The fraction of circadian-regulated genes is indicated for each group (see also Tables S2 and S3). A total of 57% of auxin-induced genes show peak expression during a 4-h window in the middle of the subjective day, compared to 22% for all circadian-regulated genes. This ratio increases to 86% for genes highly induced by auxin (≥6-fold).

Using gene expression profiling and previously described methods [12], we identified over 1,600 nuclear-encoded genes with circadian fluctuations in mRNA abundance (Table S1). Visual inspection of the data suggests that this may be an underestimate; however, this perhaps conservative estimate of circadian-regulated genes amounts to over 10% of expressed genes and is broadly consistent with previous studies [12–15]. Like circadian-regulated genes in most other organisms studied, all phases of the 24-h cycle are well represented (Figure 1A) [16,17]. When genes were clustered by function or pathway, we found several groups with an overrepresentation of circadian-regulated genes. Strikingly, genes involved in auxin signaling were disproportionately circadian regulated (*p* = 5.0 × 10^−04^) (Table S2). This overrepresentation of clock-regulated genes was not seen with the signaling components of any other hormone pathway (Table S2; unpublished data). Here, we present studies examining intersections between the clock and auxin pathways; a more global analysis of the array data will be presented elsewhere.

Interestingly, we found that all steps of auxin signaling, from production to response, had one or more genes with clock-regulated expression (Figure 1B and 1C; Table S3). The rhythmic expression patterns of auxin-signaling genes are shown in Figure 1C alongside a schematic of the auxin-signaling pathway (Figure 1B). We found two genes implicated in de novo auxin biosynthesis [31] and two auxin efflux carriers [39–41] to be subject to circadian regulation, the latter result having been reported previously [12]. Several genes encoding enzymes that inactivate auxins by conjugation to amino acids, IAA-amido synthetases [42], were coexpressed during the subjective day. Clock regulation of these transcripts may cause the previously reported circadian regulation of both free and conjugated IAA levels [21].

Examining genes implicated directly in auxin signal transduction, we found expression of one of the putative TIR1/AFB-like auxin receptors, AFB5, to be rhythmic with a broad peak around subjective dusk and into the night. We also found that genes encoding transcriptional regulators of the auxin response were clock regulated. Negative regulators of auxin responses, the *Aux/IAA* genes [43], showed peak expression during the subjective day, the same phase as the auxin-inactivating IAA-amido synthetase genes. In contrast, the transcriptional regulators mediating auxin responses (the ARFs [37]) showed an opposite phase of expression, with peak levels occurring during the subjective night. Since Aux/IAA proteins bind to and inhibit ARF function [43], this antiphasic relationship between these transcriptional regulators suggests that the clock may modulate auxin responses.

**Figure 2:**
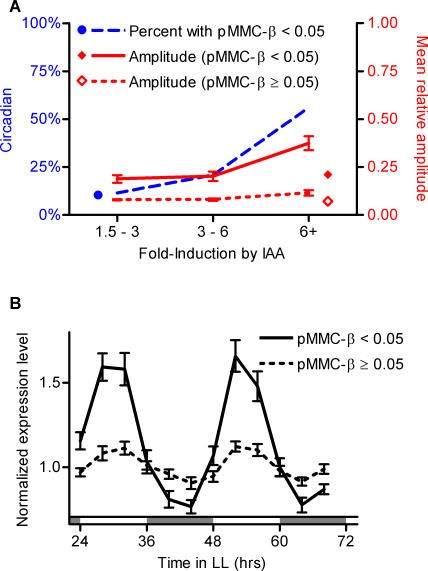
Correlation between Auxin-Responsiveness and Circadian Regulation (A) Relationship between degree of induction by auxin [36] and circadian regulation is presented. Genes were classified on the basis of responsiveness to auxin (x-axis), and the percent with clock-regulated gene expression (pMMC-β < 0.05) was plotted in blue on left y-axis; we found a significant correlation between fold induction and percent rhythmicity (χ^2^ test: *p* = 2.3 × 10^−06^). A closed blue circle (left) represents the percent of all expressed nuclear-encoded genes that are circadian regulated. There is a strong correlation between fold induction by IAA and relative amplitude of circadian-regulated genes (pMMC-β < 0.05) and a weak correlation for noncircadian genes (1 > pMMC-β ≥ 0.05), plotted in red on the right y-axis (circadian/IAA: *r*_s_ = 0.680, *p* = 4.1 × 10^−07^; noncircadian/IAA: *r*_s_ = 0.182, *p* = 1.4 × 10^−02^). Indeed, there are significant differences in relative amplitude of circadian genes between highly IAA-induced and other IAA-induced genes (t-tests: (6+) versus (3 − 6), *p* = 7.9 × 10^−04^; (6+) versus (1.5 − 3), *p* = 2.2 × 10^−04^). Discrete data points on the right represent the mean relative amplitude of all expressed nuclear-encoded genes that are circadian regulated (closed red diamond) and noncircadian (open red diamond). (B) Mean normalized microarray expression data for highly auxin-induced genes are presented [36]. Genes induced by auxin >6-fold have been classified as circadian regulated (pMMC-β < 0.05 and 19 ≤ period ≤ 29 h) or not (pMMC-β > 0.05 and/or period ≤ 19 or ≥ 29 h). Error bars represent the standard error of the mean.

Given the extensive circadian control of auxin-signaling genes, we examined the frequency of circadian expression for genes previously reported to be regulated by auxin [36,38]. We found that many more auxin-induced genes were clock regulated than expected by chance (*p* = 1.0 × 10^−04^) (Tables S2 and S3). Interestingly, we found a significant correlation between fold induction [36] and percent rhythmicity (Figure 2A). Over half of the genes highly induced by auxin are also circadian regulated. This represents a 5-fold enrichment over what would be expected by chance and more than four times that seen for genes with low auxin inducibility. Furthermore, most of these auxin- and clock-regulated genes show peak expression during a 4-h window in the middle of the subjective day (Figures 1C and S1) instead of demonstrating the broad distribution of phases observed for circadian-regulated genes in general (Figures 1A and S1). Inspection of the average expression patterns of genes that are highly induced by auxin but do not pass our *p*-value cutoff for classification as clock regulated suggests that some of these genes are in fact circadian regulated. As shown in Figure 2B, these genes on average appear to be rhythmically expressed with the same phase as highly auxin-induced genes that we do identify as circadian, suggesting that even more than 56% of highly auxin-induced genes are also clock regulated. In addition, we found a significant correlation between amplitude of cycling and degree of auxin inducibility (Figure 2A). The mean relative amplitude of the rhythms from the highly induced set of genes was nearly double that of the other two groups of auxin-induced genes (Figures 2A and S2).

We next examined circadian regulation of genes that are induced in response to the hormone brassinolide, since there is overlap between genes induced by this hormone and auxin [36]. In contrast to auxin-responsive genes, we found that genes induced by brassinolide, but not induced by auxin, were no more likely to be clock regulated than expected by chance (Table S2). Nor was there a correlation between responsiveness to brassinolide and either percent rhythmicity (*p* = 0.48) or circadian amplitude (*p* = 0.99). Furthermore, we saw no skewing of those brassinolide-induced genes that are circadian regulated towards a particular phase of peak expression as we do for those that are auxin induced (Figure S1; Table S4). These data, contrasted with the significant circadian regulation seen with auxin-signaling components, suggest that auxin but not brassinolide signaling is specifically regulated by the circadian clock.

### High Concentrations of Auxin Affect Circadian Rhythms, but Auxin Does Not Reset the Clock

**Figure 3:**
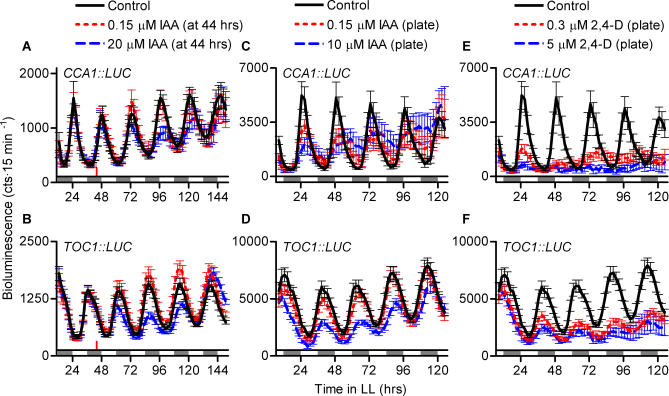
Auxin Affects Rhythmic Expression of Clock Genes in a Dose-Dependent Manner Average luciferase activity of *CCA1*::*LUC* (A, C, E) and *TOC1*::*LUC* (B, D, F) plants (*n* = 9–12) with standard error of the mean in response to exogenous auxin is presented. Seedlings were sprayed with the indicated concentrations of IAA after 44 h in continuous light (red tick mark) (A) and (B), or 1–2 h prior to the start of imaging, were transferred to growth medium containing the indicated concentrations of IAA (C) and (D) or 2,4-D (E) and (F). See Table S5 for statistical analyses. Plants were entrained for 6 d in 12-h white light/12-h dark photoperiods before being imaged in constant red light. (C) and (D) and (E) and (F) show representative data from three and two independent experiments, respectively.

Since auxin controls diverse processes [44], and there are many examples of clock outputs feeding back upon the central circadian oscillator [45–47], we examined the effects of exogenous auxin on circadian rhythms. We monitored bioluminescence rhythms of auxin-treated plants expressing the firefly luciferase gene *(LUC)* driven by the promoters of the central clock genes *CIRCADIAN CLOCK ASSOCIATED 1* (*CCA1*) and *TIMING OF CAB EXPRESSION 1* (*TOC1*) (Figure 3) [48]. We found that an acute IAA treatment administered before subjective dawn, the time at which the abundance of free IAA has been shown to be at trough levels [21], had no effect on the phase of expression of these reporter genes (Figure 3A and 3B). We also saw no phase resetting when IAA was applied at other times of day (unpublished data), suggesting that exogenous auxin is not able to set the phase of the clock. However, acute application of relatively high levels of IAA (20 μM) did cause a slight lengthening of free-running period (Table S5). We also examined the effects of acute IAA application to plants expressing luciferase under the control of promoters of the central clock-associated gene *GIGANTEA (GI)* [49] and clock output genes *CAB2, COLD-CIRCADIAN RHYTHM-RNA BINDING 2 (CCR2),* and *EARLY FLOWERING 3 (ELF3)* (Figure S3; Table S5) [46,50,51]. Similar to *CCA1*::*LUC* and *TOC1*::*LUC,* we found modest effects on free-running period length of *CAB2*::*LUC* and *GI*::*LUC* following an acute treatment of 20 μM IAA (Figures 3A, 3B, S3A, and S3D; Table S5). Consistent with a recent study [52], we found that the rhythmic amplitude of some reporters *(ELF3*::*LUC, GI*::*LUC,* and *TOC1*::*LUC)* showed a slight but significant reduction after treatment with 20 μM IAA. This reduction in amplitude was transient, lasting from two to three days after auxin application (Figures 3B, S3C, and S3D; Table S5).

Since acute auxin treatment caused a transient decrease in rhythmic amplitude, we next examined the effects of a prolonged treatment of exogenous auxin on the plant circadian clock. We transferred seedlings to growth medium containing either the natural auxin IAA or the artificial auxin 2,4-dichlorophenoxyacetic acid (2,4-D). Seedlings were maintained in monochromatic red light, a condition that minimizes photodegradation of IAA [53]. Both high concentrations of IAA (10 μM) and all tested concentrations of 2,4-D (0.3 μM and 5 μM) generally caused a slight lengthening of free-running period and a marked reduction in rhythmic amplitude (Figures 3C–3F and S3E–S3L; Table S5). Prolonged treatment with 0.15 μM IAA also decreased the rhythmic amplitude of some circadian markers (Table S5). Likewise, an acute treatment of 20 μM 2,4-D or prolonged treatment with another synthetic auxin, 5 μM 1-naphthaleneacetic acid (NAA), had similar effects on the period and amplitude of *CCR2*::*LUC* rhythms (unpublished data).

It is unclear whether auxin signaling feeds back upon the clock in a biologically relevant manner or if rhythms were indirectly affected by the toxicity of auxin treatments; the latter possibility is highlighted by the fact that 2,4-D is the most widely used herbicide worldwide [54]. Therefore, to avoid complications stemming from auxin's potentially indirect influence on rhythms, most of the subsequent experiments were performed using a low dose of auxin that had minimal or no effect on rhythmic expression of clock components and output genes.

### Auxin-Mediated Transcriptional Responses Are Circadian Regulated

**Figure 4:**
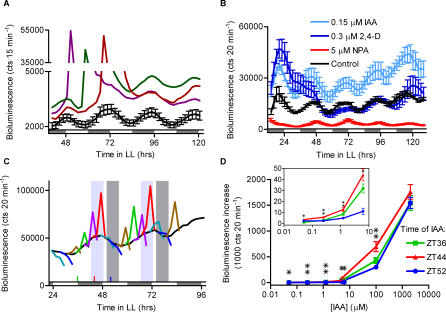
Native Auxin Signaling and Sensitivity to Exogenous Auxin Are Circadian Regulated (A) eDR5::*LUC* bioluminescence with standard error of the mean is shown for T1 plants in continuous light (black trace with error bars; *n* = 28; circadian and expression ≥5% above background). Data are also shown for eDR5::*LUC* T1 plants that were sprayed with 20 μM IAA at ZT47 (purple line), ZT55 (green line), and ZT65 (dark red line) (*n* = 32, 34, and 34; expression ≥5% above background prior to treatment). The y-axis has been split in order to better visualize both the circadian and the auxin-responsive expression of eDR5::*LUC*. (B) eDR5::*LUC* rhythms in the presence of exogenous auxins (IAA and 2,4-D) and an auxin transport inhibitor (NPA) are shown. Bioluminescence from the apex of each seedling was measured (*n* = 3–18). (C) Circadian gating of auxin sensitivity is presented. Groups of eDR5::*LUC* plants (*n* = 8) were treated with 0.15 μM IAA at 4-h intervals. Bioluminescence levels at 1 h prior to treatment and 1, 3, and 5 h after treatment are shown in various colors for each auxin application. Data from treated samples have been normalized such that the bioluminescence level of the pretreatment time point matches that of the control, shown in black (i.e., all values for a particular treatment have been divided by the pretreatment value and multiplied by the control value that corresponds to the pretreatment time point). The color-coded tick marks above the x-axis correspond to the times of auxin application data in Figure 4D. Areas shaded light- and dark-gray correspond to the 6-h periods in Figure 5A during which exogenous auxin promotes or has no effect, respectively, on hypocotyl elongation. (See Figure S5 for an alternate presentation of the data from Figure 4C, see Figure S6 for this data plotted adjacent to the data from Figure 5A.) (D) eDR5::*LUC* plants were sprayed with the indicated concentrations of IAA at different times of day as described for (C). Presented are the differences in bioluminescence 3 h after IAA treatment compared to the control plants. The lower range of doses is also shown as an inlay. One or two asterisks indicate that the response at ZT44 is significantly (*p* < 0.05) greater than the responses at one or both of the other time points, respectively. Datasets are color coded to match the tick marks above the x-axis in Figure 4C. (A–C) and (D) show representative data from two and three independent experiments each, respectively.

The congruence between auxin- and clock-regulation of gene expression, along with the conspicuous circadian regulation of auxin-signaling genes, suggested that there might be circadian regulation of auxin signal transduction. To investigate this possibility, we devised a new tool for conveniently monitoring temporal regulation of auxin transcriptional responses in individual plants. We generated transgenic plants expressing the firefly luciferase gene under the control of an enhanced version of the auxin-responsive promoter DR5, a well-characterized synthetic promoter [55]. The original DR5 promoter has been widely used to drive the expression of β-Glucuronidase and green fluorescent protein to monitor the spatial domain of auxin responses. This DR5 promoter consists of seven repeats of an 11-bp sequence derived from the AuxRE found upstream of the auxin-induced soybean *GH3* gene [55]. Since luciferase activity in DR5::*LUC* seedlings was too low to monitor (unpublished data), we increased the number of tandem repeats of the core element from seven to 13 to generate an enhanced DR5 reporter construct (eDR5::*LUC*). Auxin-responsive bioluminescence can be easily detected in plants transgenic for this reporter construct. All eDR5::*LUC* transformants that were tested exhibited dramatic induction of luciferase activity in response to an acute application of 20 μM IAA, with luciferase activity declining to near basal levels approximately 24 h after treatment (Figure 4A). In contrast, plants expressing luciferase under the control of a mutated form of the eDR5 promoter, m3,4-eDR5, did not show an increase in activity after auxin application (unpublished data), consistent with previous reports [55].

**Figure 5:**
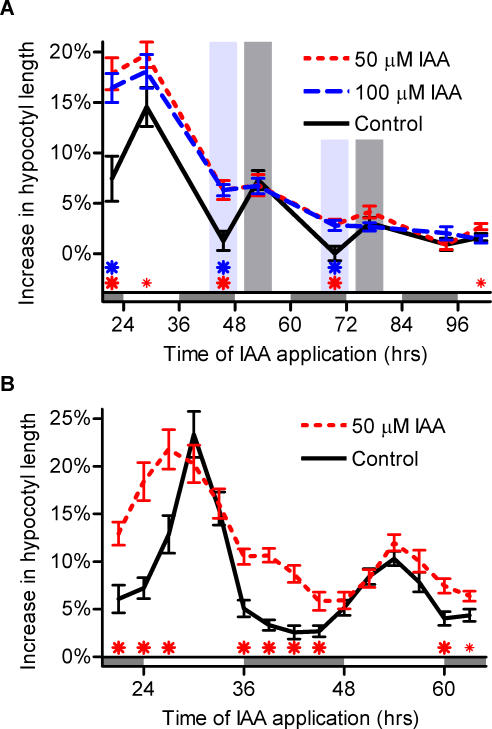
Auxin-Induced Hypocotyl Elongation Is Gated by the Circadian Clock Hypocotyl elongation of free-running plants in response to auxin treatment was monitored twice a day for 4 d (A) or at 3-h intervals over 2 d (B). Plants were entrained for 3 d in 12-h white-light/12-h dark photoperiods and then transferred to constant red light. The time of auxin or mock treatment is graphed on the x-axis and the percent increase in hypocotyl length observed over the subsequent 6 h is indicated on the y-axis. Data points are plotted in the middle of each 6-h treatment. Asterisks on the x-axis indicate time points at which hypocotyl elongation of auxin-treated seedlings was significantly greater than in the controls (t-tests: small asterisk if *p* < 0.05, large asterisk if *p* < 0.01). (A) Areas shaded light- and dark-gray correspond to the 6-hr periods during which exogenous auxin promotes or has no effect, respectively, on hypocotyl elongation. (A) Shows representative data from two independent experiments. (See Figure S6 for a direct comparison of Figures 4C and [A]).

We next examined temporal regulation of luciferase activity in the absence of exogenous auxin. Of those T1 seedlings expressing eDR5::*LUC* with mean bioluminescence levels at least 5% above background, over 80% expressed eDR5::*LUC* in a circadian manner. Importantly, these seedlings showed a consolidated phase of peak activity around subjective dawn (Figure 4A). In contrast, only 12% of the visible m3,4-eDR5::*LUC* plants (mean bioluminescence levels at least 5% above background) exhibited circadian patterns of bioluminescence, and none were dawn phased like eDR5::*LUC* (Table 1) (unpublished data). Circadian regulation of luciferase activity in these rare plants is likely due to random insertion of the transgene in the promoters of clock-regulated genes. These data demonstrate that auxin signaling is indeed under circadian control, as suggested by the predominance of clock regulation among highly auxin-induced genes.

**Table 1:**
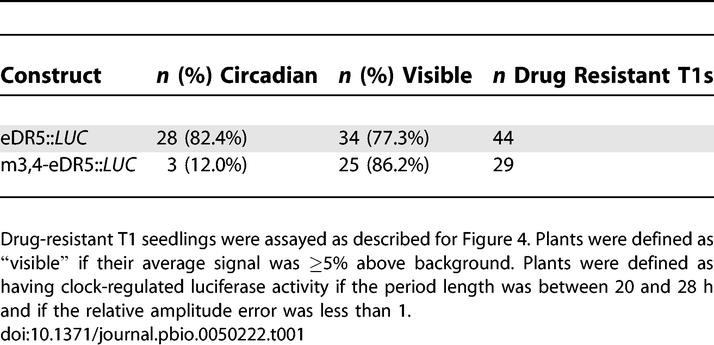
eDR5::*LUC* Expression Is Circadian Regulated

### Circadian Control of Auxin-Regulated Gene Expression Does Not Depend upon Rhythmic Auxin Biosynthesis or Transport

Previous reports have indicated that levels of active IAA are under circadian regulation in *Arabidopsis* [21]. We therefore investigated whether circadian regulation of luciferase activity in eDR5::*LUC* plants was affected by exogenous auxin. Acute treatment with 20 μM IAA caused a transient increase in luciferase activity in these plants, but as was generally true for other clock outputs, no change in circadian phase or amplitude was observed (Figure 4A). We next tested the effects of prolonged treatment with a natural auxin by transferring eDR5::*LUC* plants to media containing IAA. Luciferase activity was elevated relative to control plants (Figure 4B), demonstrating that these plants were taking up active hormone throughout the experiment. However, rhythmic luciferase activity was maintained, and normalization revealed there was no change in the amplitude of rhythmic gene expression (Figure S4). We also examined eDR5::*LUC* expression in plants grown on media containing the extremely stable [56] synthetic auxin 2,4-D. Rhythmic luciferase activity persisted under these conditions (Figures 4B and S4). Slight effects on free running period and rhythmic amplitude were observed, similar to what was seen for other circadian markers (Figures 3E, 3F, and S3I–S3L; Table S5). Rhythmic auxin signaling thus persists in the presence of exogenous auxin, suggesting that these rhythms can be controlled by processes downstream of auxin synthesis.

The site of auxin synthesis is often not the site of auxin action [31]. The observation that the auxin efflux carriers PIN-FORMED 3 (PIN3) and PIN7 show circadian regulation of gene expression suggested that rhythmic auxin transport might be essential for the observed rhythmic transcriptional responses. To test this possibility, we grew eDR5::*LUC* plants on media containing *N*-1-naphthylphthalamic acid (NPA), an inhibitor of the auxin transport machinery. Although NPA alters the location of primary sites of eDR5::*LUC* expression (as previously reported for DR5::*β-GLUCURONIDASE* plants [57,58]), rhythms are still detected (Figure 4B). NPA caused an ∼2-h delay in the phase of eDR5::*LUC* rhythms and a decrease in expression levels, but the amplitude of the rhythm did not decrease (Figure S4). Furthermore, transport of 2,4-D is not dependent upon PIN proteins [31,59], so eDR5::*LUC* rhythms in the presence of 2,4-D (Figure 4B) are likely independent of rhythmic PIN activity. Likewise, rhythms were still detectable in the presence of NAA (unpublished data), an extremely stable [56] synthetic auxin able to bypass auxin influx carriers by diffusing into cells [31,59]. Our data therefore suggest that neither rhythmic auxin synthesis nor transport is required for circadian regulation of auxin signaling.

### Transcriptional Responses to Auxin Are Gated by the Circadian Clock

Many responses that are under both circadian and environmental regulation are also gated by the clock [11,60]. The apparent time-of-day-dependent differential responses to 20 μM IAA observed in Figure 4A suggested that this may also be the case for auxin signaling. Given this preliminary result and the circadian regulation of both auxin-signaling components and auxin-responsive genes, we wished to discover whether the sensitivity of the plant to exogenous auxin might indeed be controlled by the clock. We therefore applied low doses of IAA to separate groups of free-running eDR5::*LUC* seedlings at 4-h intervals over the course of several days and monitored the degree of induction of luciferase activity after each application. When given during the subjective day, IAA treatments caused little or no increase in luciferase activity and even on occasion a decrease, although this latter observation was not consistently reproducible (Figures 4C and S5). Thus plants show minimal responsiveness to exogenous IAA at times when eDR5::*LUC* expression in control plants is decreasing. At subjective dusk, the time at which nontreated eDR5::*LUC* levels begin an upward trend from trough to peak, the sensitivity to exogenous IAA increased as well. Peak IAA responsiveness and peak levels of eDR5::LUC activity in untreated plants thus coincided just before subjective dawn. The gating of eDR5::*LUC* induction appears to be slightly less robust towards the end of the time course. An additional day of monitoring revealed that in these older plants auxin responses seem more sustained at all time points, but that gating persists (unpublished data). This diminished gating after an extended time in constant conditions is similar to what was reported for the circadian gating of light-induced *CAB2*::*LUC* expression [11].

We next wished to determine whether the observed gating was consistently observed only at low IAA concentrations. We therefore applied a wide range of auxin concentrations, from 0.15 μM to 10 mM IAA, to plants at three different times during the subjective day and night. At IAA concentrations between 0.15 μM to 100 μM, plants treated during the late subjective night (Zeitgeber Time 44 [ZT44]) were more responsive than those treated at other times. However, at the very high dose of 2 mM IAA, differential sensitivity was no longer observed (Figure 4D). There is no additional induction of eDR5::LUC activity when auxin treatments are increased from 2 mM to 10 mM (unpublished data), suggesting that at these high concentrations this auxin response is saturated. Therefore, both endogenous auxin signaling and plant responses to a wide range of concentrations of exogenous auxin are regulated by the circadian clock.

### Growth Responses to Auxin Are Gated by the Circadian Clock

We next wanted to investigate whether the circadian control of auxin signaling would be manifested in other types of outputs. Therefore, we decided to focus on plant growth, a process subject to both auxin and circadian regulation. Auxin has long been implicated in plant growth, while both hypocotyl and internode elongation have more recently been shown to be circadian regulated [21,22]. Since we had been working with seedlings up to this point, we examined circadian control of auxin-induced growth in the hypocotyl.

Entrained plants were transferred to continuous red light, and hypocotyl elongation was examined in control- and IAA-treated seedlings over 6-h periods for multiple days. Rhythmic elongation with a peak in the mid-to-late subjective day was observed in control plants (Figure 5), consistent with previous reports [22]. Rhythmic amplitude decreased over successive days as the hypocotyls approached their maximum length. In general, we found that elongation could be enhanced when plants were treated with exogenous IAA during the subjective night, but that no additional growth was seen when plants were treated during the subjective day (Figure 5). Just as the amplitude of hypocotyl elongation rhythms diminished through development, the additional growth responses to exogenous IAA became minimal or nonexistent over time (Figure 5A). Interestingly, exogenous auxin caused maximal stimulation of both plant growth and eDR5::LUC activity at the same time, during the subjective night (see Figure S6 or compare light-gray shaded areas in Figures 4C and 5A). In contrast, the time of peak hypocotyl elongation in untreated control plants occurred later in the subjective day (Figure 5). The first day of data is not shaded in Figures 4C and 5A because we have observed that following the transition from entraining conditions to growth in constant light, hypocotyl growth rhythms are unstable for up to a day and a half [61]. This is likely due to a complex interplay between light signaling and the circadian clock on the regulation of hypocotyl elongation. Likewise, the fourth and final day is not shaded since hypocotyl elongation rhythms have diminished because of cessation of hypocotyl growth.

The limited temporal resolution of the data in Figure 5A left open the possibility that there is a maximal hypocotyl elongation rate that (1) is attained once a day in control seedlings and (2) can be reached at any time of day if plants are treated with exogenous auxin. To address this possibility, we performed a time-course analysis with better temporal resolution (Figure 5B). We found that hypocotyl elongation in response to exogenous auxin diminished as subjective dawn approached, reaching a growth rate equivalent to the controls at ZT48. Notably, this growth rate is lower than that observed in both control and IAA-treated plants between hours 51 and 57. Thus auxin treatment around subjective dawn (ZT48) was not sufficient to increase elongation to the levels seen either a few hours earlier or later. This result indicates that the differential growth response to auxin at different times of day represents bona fide gating by the circadian clock. Circadian gating of sensitivity to auxin therefore extends from auxin-regulated gene expression to auxin-regulated growth responses, suggesting that these processes are causally linked.

## Discussion

Using genome-wide transcriptional profiling, we found that over 10% of genes expressed in *Arabidopsis* seedlings are circadian regulated at the level of transcript abundance. This is likely an underestimate given our observation of apparent rhythms in the expression of highly auxin-induced genes that we have classified as noncircadian. Indeed, another recent global examination of clock-regulated gene expression estimated that at least 16% of *Arabidopsis* genes show circadian regulation of transcript abundance [14]. We were intrigued by the preponderance of circadian-regulated genes involved in auxin signaling. Members of three families of auxin-signaling components have been shown to have relatively short half-lives [62–64]. Thus transcript abundance for these gene families is likely a good indicator of their protein levels. The possibility that auxin signaling might be under circadian control was bolstered by our finding that over 50% of highly auxin-responsive genes are also rhythmically expressed. Using multiple repeats of a well-characterized synthetic auxin-responsive motif to drive a reporter gene, we discovered that plant responses to endogenous auxin are indeed clock regulated. Further, we found that the circadian clock gates plant transcriptional and growth responses to exogenous auxin. Thus, we have described an exciting and unsuspected link between clock and auxin signaling, two important, far-reaching, and intensively studied pathways.

The activity of the synthetic auxin-responsive promoter DR5 is commonly used as an estimate of active auxin levels. The circadian nature of auxin signaling suggests caution is required when using DR5 activity or other auxin-controlled processes in this manner. It might be most appropriate to think of DR5 as an indicator of the state of auxin signaling rather than an indicator of free auxin levels. Interestingly, we observed peak activity of eDR5::LUC around subjective dawn, about 6 h before peak expression of most auxin-induced genes. This modest phase discrepancy may be due to inherent differences between natural and pared-down synthetic promoters. For example, endogenous AuxREs tend to be composite sequences with a “constitutive element” adjacent to the motif on which the synthetic DR5 promoter was based [65].

Since many auxin-signaling components are clock regulated at the transcriptional level, it is not obvious where the clock- and auxin-signaling pathways intersect. One possibility is that this integration occurs at the promoters of individual genes. However, this seems unlikely for several reasons. First, the auxin-responsive minimal promoter eDR5 drives circadian regulation of the luciferase gene, whereas m3,4-eDR5 (with two nucleotides altered per each 11-bp repeat) has lost both auxin responsiveness and circadian regulation. Second, we found a significant correlation between high auxin inducibility and high-amplitude circadian rhythms. Finally, auxin-regulated genes that are also under circadian control show a consolidated phase of peak expression during the subjective day. All of these lines of evidence suggest that the auxin signal transduction pathway itself is regulated by the clock.

To further delineate where the clock and auxin pathways interact, we took a pharmacological approach. Our findings that rhythmic eDR5::*LUC* expression persists in plants grown on media containing exogenous IAA and that transcriptional response to even high doses of IAA are gated by the clock suggest that rhythmic auxin synthesis is not required for clock regulation of auxin signaling. This is consistent with previous studies showing that rhythmic internode elongation was rescued in decapitated floral stems when active auxin was applied to the cut surface [21]. We also observed rhythmic luciferase activity when plants were grown on media containing the synthetic auxins 2,4-D or NAA. Given that 2,4-D is a poor substrate for the enzymes that conjugate IAA to amino acids [42], many of which show clock-regulated gene expression, this suggests that rhythmic conjugation may also be unnecessary for circadian auxin signaling. NAA and 2,4-D do not depend upon the usual auxin influx and efflux carriers, respectively [31,59], suggesting that rhythmic auxin transport is also not required for the observed rhythms in transcriptional responses. Indeed, rhythms persisted when plants were grown on media containing the auxin transport inhibitor NPA. Therefore it seems likely that clock modulation of the auxin-signaling pathway occurs at the level of auxin perception and/or signaling.

Although our data suggest that rhythmic biosynthesis, transport, and conjugation are not individually required for establishing auxin-signaling rhythms, clock regulation of these steps might act to reinforce rhythms generated by some downstream component. Alternatively, it is possible that circadian regulation of no single step in the auxin-signaling pathway is required for circadian auxin signaling. Rather, clock regulation of multiple steps might work together to establish and maintain circadian auxin signaling, similar to what has been observed for clock regulation of the production of plant scent compounds [66]. Clarification of this point awaits the detailed phenotypic analysis of various auxin-signaling mutants.

We also found that auxin promotes growth in intact plants in a time-of-day–specific manner. Although auxin was first identified over a hundred years ago on the basis of its ability to promote growth in decapitated plants and plant sections, extensive studies with intact wild-type plants have found that exogenous auxin usually inhibits, rather than enhances, hypocotyl growth [67–69]. In our hypocotyl elongation studies we treated growing seedlings with exogenous auxin, whereas most researchers assaying the effects of auxin on intact wild-type *Arabidopsis* plants germinate seeds on auxin-containing media [67,69]. To determine whether this might affect plant growth responses to the hormone, we compared the effects of auxin treatments given before and after germination. Consistent with previous reports, seeds germinated on media containing auxin show a clear inhibition of hypocotyl elongation after 5 d of growth (Figure S7A). However, treatment of already-growing seedlings with auxin promotes hypocotyl elongation over a wide range of treatment times (Figure S7B), suggesting that developmental stage, not treatment duration, dictates the effect of auxin on hypocotyl elongation. This is consistent with our observation that auxin responses are more sustained in older seedlings (unpublished data) and highlights the complex interaction of temporal, spatial, and developmental parameters on auxin signaling and responses.

Auxin-signaling pathways are well known to undergo negative feedback regulation, leaving open the possibility that circadian gating of auxin signaling is also modified by negative feedback. Many other processes both promoted by an external stimulus and regulated by the endogenous clock show circadian gating; often the response is modulated such that the pattern of responsiveness mirrors the pattern of endogenous cycling [11,60]. However, the acute induction of eDR5::*LUC* expression in response to exogenous auxin shows an abrupt decline in the subjective late night/early morning, creating a sawtooth-like variation in sensitivity not seen in eDR5::*LUC* rhythms in untreated plants. Intriguingly, transcript levels of IAA-amido synthetase and Aux/IAA genes, the products of which result in inactivation of and decreased sensitivity to IAA, begin to increase at the time when sensitivity to exogenous IAA abruptly drops. This raises the possibility that gated induction of these genes by the clock might cause the observed abrupt decrease in auxin sensitivity early in the subjective day. Notably, early morning is also the time of day when plants show the least auxin-induced hypocotyl elongation. Clock regulation of multiple aspects of auxin signaling may thus modulate plant responses to exogenous auxin.

Hypocotyl elongation has previously been shown to be under circadian regulation with the peak rate of growth occurring around subjective dusk when plants are maintained in constant light [22]. Our data suggest that rhythmic sensitivity to auxin does not cause the growth rhythms seen in constant light, since in these conditions peak responses to auxin occur during the subjective night while most growth occurs during the mid- and late-subjective day. However, we found that most auxin-induced genes show peak clock-regulated expression in the subjective afternoon, not long before the observed peak in hypocotyl growth rate. This suggests that normal rhythmic hypocotyl elongation may be influenced by circadian auxin signaling, which can be readily tested when more is known about the basis for the clock's influence over auxin signaling. Identifying the underlying mechanisms behind clock-regulated growth processes is proving to be a challenging task [70]. It is likely that rhythmic growth is controlled by a complex interplay of multiple signaling networks perhaps including auxin, ethylene, circadian, and light-signaling pathways, among others.

Auxin and the circadian clock both play pervasive roles in plant growth, development, and responses to the environment. Previous studies have emphasized the role of auxin in spatial regulation [27,28,71]. Our finding that auxin signaling is regulated by the circadian clock raises the possibility that there is also a temporal component in the effects of auxin on plant growth and development. It will be very exciting to determine the extent to which auxin-mediated responses such as tropisms and organ formation are also regulated by the circadian clock. Such insights will help us better understand how the clock helps plants cope with their ever-changing environment.

## Materials and Methods

### Microarray analysis.

*CCR2*::*LUC* (Col-0 ecotype) seeds were vapor-phase sterilized (100 ml bleach with 3 ml HCl) for 10 h prior to being sown on filter papers on MS agar plates containing 3% sucrose and 0.8% agar. Seeds were stratified at 4 °C for 4 d before transfer to a growth chamber (22 °C). Seedlings were entrained in 12-h white light (light source was cool white fluorescence tubes; fluence rate ∼120 μmol m^−2^ sec^−1^)/12-h dark cycles for 7 d before being released into free-running conditions of continuous white light at 22 °C. Starting at subjective dawn of day 9, tissue was harvested every 4 h over the course of the next 44 h. Following standard protocols (previously described in [12]), labeled cRNA targets were prepared from total RNA and hybridized to oligonucleotide-based arrays with probe sets corresponding to over 22,000 *Arabidopsis* genes, nearly the entire genome. Approximately 69% of these genes are expressed in our samples. To be considered “expressed,” the gene must be called “present” by MAS 5.0 Software (Affymetrix, http://www.affymetrix.com) for at least four of the 12 time-points. To identify expressed genes whose transcript abundance fluctuates with a period of ∼24-h, the dChip-derived Model-Based Expression Index [72] of each probe set was fit to cosine waves of defined period and phase and the significance of fit determined by empirical testing [73]. This method has been widely used to analyze circadian microarray datasets [12,16,17]. Transcript abundance of 1,610 nuclear-encoded genes, corresponding to 10.4% of genes with detectable expression levels, had a greater than 95% probable correlation (pMMC-β < 0.05) to a cosine wave with a period between 19 and 29 h; these genes were called “circadian regulated.” The relative amplitude is defined as the ratio of β (a measure of absolute amplitude) to mean Model-Based Expression Index of the 12 time points. The heat map display in Figure 1A was generated using Prism [74], a Web-based genomic data visualization program (http://noble.gs.washington.edu/prism).

### Luciferase imaging.

The eDR5::*LUC* construct was generated by inserting 13 copies of the 11-nucleotide DR5 core element (instead of the seven copies of the core element used in previous DR5 reporters) into the BamHI/XhoI sites of pAtM-NOS [75]. The m3,4-eDR5::*LUC* construct contains 13 copies of a mutated core DR5 element repeat containing two mutated nucleotides [55]) inserted into the SacI/XhoI sites of pAtM-NOS [75]. For *GI*::*LUC,* a 1.3-kb region upstream of the *GI* coding sequence was PCR amplified (PCR primers: CAC AAT CAC GgA TCg TAT GGA G and GAC ATC AAA aGc TTC GGG AAA [inserted endonuclease recognition sites are underlined, and nucleotide changes are in lowercase]) and inserted into the BamHI/HindIII sites of pAtM-DΩ. Transgenic plants were generated as previously described [75]. Plants expressing luciferase driven by promoters from *CAB2* [50], *CCA1* [76], *CCR2* [51], *ELF3* [46], and *TOC1* [77] have been previously described. Except where indicated, seedlings were grown on MS medium (Gibco BRL/Invitrogen, http://www.invitrogen.com) with 0.8% agar and 3% sucrose with entrainment in 12-h white light (∼50 μmol m^−2^ sec^−1^)/12-h dark cycles for 7 d before being sprayed with 1.5 ml of 3 mM D-luciferin (Biosynth AG) in 0.1% Triton X-100 and released into free-running conditions of continuous red light (∼50 μmol m^−2^ sec^−1^) for imaging. Seedlings were assayed for bioluminescence by acquiring images every 2 h with exposure times of 15 min (Figures 3, 4A, and S3) using an ORCA II ER CCD camera (Hamamatsu Photonics, http://www.hamamatsu.com) or 20 min (Figures 4B–4D, S4, and S5) using a DU434-BV CCD camera (Andor Technology, http://www.andor.com). Background subtraction was performed for all luciferase-imaging experiments. Fourier transform nonlinear least squares analysis [78] was performed to estimate period, phase, amplitude, and relative amplitude error of rhythmic luciferase activity. Rhythms were considered circadian if the period length was between 20 and 28 h and if the relative amplitude error was less than 1. IAA (Sigma, I-2886, http://www.sigmaaldrich.com) and 2,4-D (Sigma, D-7299) were dissolved in ethanol and NPA (Chem Service, PS-343, http://www.chemservice.com) was dissolved in DMSO. Acute IAA treatments were given as a spray (in 0.1% Triton X-100) with a volume of 1.5 ml. Controls for IAA and 2,4-D treatments are presented and included 0.1% Triton X-100 with the appropriate amount of ethanol (up to 0.0033%). The control for NPA treatment (DMSO at a final concentration of 0.0005%) did not affect rhythmic eDR5::*LUC* expression (unpublished data).

### Hypocotyl growth assay.

After stratification at 4 °C, Col-0 seeds were sown on filter papers on MS agar plates containing 3% sucrose and 0.8% agar and transferred to entraining conditions of 12-h white light (∼50 μmol m^−2^ sec^−1^)/12-h dark cycles for 3 d. Seedlings were then transferred to continuous red light (∼25 μmol m^−2^ sec^−1^). During the first subjective night treatments began and continued over the course of several days either twice a day (Figures 5A and S6) or every 3 h (Figure 5B). Seedlings were treated with 1 ml of an MS solution containing 50 μM IAA (in ethanol), 100 μM IAA, or ethanol (of same volume used in IAA treatments). For each time point, seedlings were transferred into a pool of 1 ml treatment solution that had been applied to fresh MS agar plates containing 3% sucrose and 0.8% agar. Seedlings were imaged immediately after application and left to grow for an additional 6 h in red light before being imaged a second time (Canon PowerShot SD500, http://www.canon.com). ImageJ (http://rsb.info.nih.gov/ij) was used to measure percent change in hypocotyl length.

## Supporting Information

Figure S1 The Phase Distributions of Hormone-Induced Circadian-Regulated Genes The phase distributions of genes solely induced by auxin (IAA-up only) and by both auxin and brassinolide (IAA-up and BL-up), but not genes only induced by brassinolide (BL-up only) [36], deviate significantly from that expected by chance. See also Table S4.
(1.2MB TIF).

Figure S2 Highly Auxin-Responsive Circadian-Regulated Genes Have Higher Amplitude Rhythms Mean normalized microarray expression data with standard error of the mean for circadian-regulated auxin-induced genes are presented [36]. The relative amplitude for rhythms of circadian-regulated highly auxin-induced (>6-fold) genes is significantly greater than that of circadian-regulated genes that exhibit intermediate (3- to 6-fold) or low levels (1.5- to 3-fold) of induction by auxin (*p* = 2.2 × 10^−04^ and *p* = 7.9 × 10^−04^, respectively).
(11.3 MB TIF)

Figure S3 Auxin Affects Rhythmic Expression of Other Clock-Regulated Genes in a Dose-Dependent Manner Average luciferase activity of *CAB2*::*LUC* (A, E, and I), *CCR2*::*LUC* (B, F, and J), *ELF3*::*LUC* (C, G, and K), and *GI*::*LUC* (D, H, and L) plants (*n* = 7–13) with standard error of the mean in response to exogenous auxin are shown. Seedlings were treated as described for Figure 3. Red tick marks on x-axes indicates the time of IAA application, 44 h (A–D). See Table S5 for statistical analyses. (E–H) and (I–L) show representative data from three and two or more independent experiments, respectively.
(25.0 MB TIF)

Figure S4 eDR5::*LUC* Rhythms in the Presence of Exogenous Auxins or an Auxin Transport Inhibitor Data presented in Figure 4B have been normalized by dividing each time point by the mean bioluminescence level for each dataset.
(6.5 MB TIF)

Figure S5 Circadian Gating of Auxin Sensitivity Data from the gating experiment presented in Figure 4C have been replotted in a non-normalized fashion. For each time series, the basal luminescence prior to auxin treatment has been subtracted from all subsequent values for that series, following previously described analysis methods [79,80]. Other details are as described for Figure 4C.
(5.9 MB TIF)

Figure S6 Distinct Auxin Responses Are Gated by the Circadian Clock at the Same Time of Day To demonstrate the congruence of times during which exogenous auxin treatments elicit transcriptional and growth responses or have little to no effect, data from Figures 4C (top) and 5A (bottom) have been plotted together. Areas shaded light- and dark-gray correspond to the 6-h periods during which exogenous auxin promotes or has no effect, respectively, on hypocotyl elongation.
(9.5 B TIF)

Figure S7 Hypocotyl Elongation during Extended Auxin Treatments Seedlings were treated with auxin for 5 d starting either prior to germination (A) or after ∼4 d of growth (B).
(A) Representative seedlings show the inhibitory effects of auxin early in development. Seedlings were germinated directly on control plates (−) or plates containing 50 μM IAA (+). After being entrained for 2 d in 12-h white light/12-h dark photoperiods, seedlings were grown for three additional days in continuous red light and then scanned (Microtek ScanMaker 8700, http://www.microtek.com). The white bar represents a length of 1 mm.
(B) An increase in hypocotyl elongation is observed when plants are treated with auxin after germination. Plants were entrained in 12-h white light/12-h dark photoperiods on plates containing no auxin for 4 d and then transferred to constant red light. After 18 h in free-running conditions, seedlings were transferred either to plates with growth media containing 50 μM IAA and sprayed with 2 ml 50 μM IAA or to control plates. Seedlings were imaged immediately after transfer (time = 0 h) and returned to red light. Seedlings were imaged 6 h later and then periodically over the next 5 d. Hypocotyl length was determined using ImageJ and plotted +/− standard error of the mean. The difference in elongation between treated and control seedlings is statistically significant (*p* < 0.02) for all time points. (A and B) show representative data from two independent experiments.
(15.4 MB TIF)

Table S1 Circadian Analysis of Microarray Time Course Affymetrix probe sets and their mappings to TAIR6 genome release, model-based expression indexes and standard errors from dChip analysis [72], and presence calls from MAS 5 (Affymetrix) analysis are shown. Results of circadian analysis (such as period, phase, relative amplitude, and pMMC-β derived using COSOPT [73]) are only shown for probe sets with pMMC-β < 1 and 18 ≤ period ≤ 30 h. A gene is considered circadian regulated if pMMC-β < 0.05 and 19 ≤ period ≤ 29 h. Genes mapping ambiguously to both nuclear- and plastid-encoded genes are classified as plastid encoded. Genes are considered to be expressed if they are deemed present in at least four of the 12 samples. Circadian phase values are calculated by taking into account both time of peak expression and period length of each rhythm (24 × [phase estimate of peak expression relative to dawn]/[period estimate]).
(13.2 MB XLS)

Table S2 Circadian-Regulated Genes Are Overrepresented in Genes Involved in Auxin Signaling and Genes Induced by Auxin For each group of genes [31,34,36,37,39,42,43], permutation testing was performed to derive *p*-values to determine significant overrepresentation of circadian-regulated genes. Significant *p*-values from 10,000 permutations are shown in bold red type.
(20 KB XLS)

Table S3 Breakdown of Auxin-Signaling Genes and Auxin-Induced Genes that Are Circadian Regulated Included are lists of auxin-related genes [31,34,36,37,39,42,43] that are: expressed and circadian; expressed, but not circadian; not expressed; and not represented on the microarray.
(59 KB XLS)

Table S4 The Phase Distribution of Auxin-Induced, but not Brassinolide-Induced, Circadian-Regulated Genes Deviates from That Expected by Chance χ^2^ tests were performed to determine whether the phase distributions of circadian-regulated genes induced by IAA and/or BL application [36] (see Figure S1) differed significantly from the phase distribution of all circadian-regulated genes. Significant *p*-values from χ^2^ tests are shown in bold red type.
(13 KB XLS)

Table S5 The Effects of Exogenous Auxin on Rhythmicity, Period Length, and Amplitude Statistical analyses of the bioluminescence data shown in Figures 3 and S3 are presented. The percentage of plants with detectable rhythms, free-running period, and relative amplitudes are displayed. Plants were defined as rhythmic if they had an estimated period between 20 and 28 h with a relative amplitude error, a measure of rhythmic robustness, less than 1 [78]. Treatments that resulted in less than 100% rhythmicity are shown in bold type. The variance-weighted mean period length in hours is shown for circadian rhythms observed for mock-treated plants, with the change in period length seen in auxin-treated plants. The mean relative amplitude is shown for circadian rhythms observed for mock-treated plants, with the change in relative amplitude seen in auxin-treated plants. Significant *p*-values from t-tests (and corresponding changes in response to auxin) are shown in bold red type.
(27 KB XLS)

### Accession Numbers

All array data have been deposited in the Gene Expression Omnibus database (GEO) (http://www.ncbi.nlm.nih.gov/geo) with accession number GSE8365.

## Abbreviations

2,4-D: 2,4-dichlorophenoxyacetic acid
AFB: auxin-signaling F-box protein
ARF: auxin response factor
AuxRE: auxin-responsive element
CAB2: chlorophyll a/b binding protein 2
CCA1: circadian clock associated 1
CCR2: cold-circadian rhythm-RNA binding 2
ELF3: early flowering 3
GI: gigantea
IAA: indole-3-acetic acid
LUC: luciferase
NAA: 1-naphthaleneacetic acid
NPA: N-1-naphthylphthalamic acid
PIN: pin-formed
TIR1: transport inhibitor response 1
TOC1: timing of cab expression 1
ZT: zeitgeber time
